# Fucoidan Reduces Secretion and Expression of Vascular Endothelial Growth Factor in the Retinal Pigment Epithelium and Reduces Angiogenesis In Vitro

**DOI:** 10.1371/journal.pone.0089150

**Published:** 2014-02-18

**Authors:** Michaela Dithmer, Sabine Fuchs, Yang Shi, Harald Schmidt, Elisabeth Richert, Johann Roider, Alexa Klettner

**Affiliations:** 1 University of Kiel, University Medical Center, Department of Ophthalmology, Kiel, Germany; 2 University of Kiel, University Medical Center, Experimental Trauma Surgery, Kiel, Germany; 3 MetaPhysiol, Essenheim, Germany; Casey Eye Institute, United States of America

## Abstract

Fucoidan is a polysaccharide isolated from brown algae which is of current interest for anti-tumor therapy. In this study, we investigated the effect of fucoidan on the retinal pigment epithelium (RPE), looking at physiology, vascular endothelial growth factor (VEGF) secretion, and angiogenesis, thus investigating a potential use of fucoidan for the treatment of exudative age-related macular degeneration. For this study, human RPE cell line ARPE-19 and primary porcine RPE cells were used, as well as RPE/choroid perfusion organ cultures. The effect of fucoidan on RPE cells was investigated with methyl thiazolyl tetrazolium – assay, trypan blue exclusion assay, phagocytosis assay and a wound healing assay. VEGF expression was evaluated in immunocytochemistry and Western blot, VEGF secretion was evaluated in ELISA. The effect of fucoidan on angiogenesis was tested in a Matrigel assay using calcein-AM vital staining, evaluated by confocal laser scanning microcopy and quantitative image analysis. Fucoidan displays no toxicity and does not diminish proliferation or phagocytosis, but reduces wound healing in RPE cells. Fucoidan decreases VEGF secretion in RPE/choroid explants and RPE cells. Furthermore, it diminishes VEGF expression in RPE cells even when co-applied with bevacizumab. Furthermore, fucoidan reduces RPE-supernatant- and VEGF-induced angiogenesis of peripheral endothelial cells. In conclusion, fucoidan is a non-toxic agent that reduces VEGF expression and angiogenesis in vitro and may be of interest for further studies as a potential therapy against exudative age-related macular degeneration.

## Introduction

Age-related macular degeneration (AMD) is the leading cause for legal blindness in the industrialized countries and, due to demographic developments, the burden of AMD will increase both as a clinical and as a socio-economical problem [1]. Factors discussed to contribute to AMD development are oxidative stress, chronic inflammation and complement activation [2]–[4]. In exudative, or wet, AMD, which is responsible for the majority of vision loss in AMD, choroidal neovascularizations (CNV) occur, in which vessels grow from the choroid into the subretinal and retinal space. These immature vessels leak into the retina, leading to vision loss or blindness [5]. For the development of CNV, the presence of vascular endothelial growth factor (VEGF) is vital [6]. Currently, no cure for wet AMD is available, but a deceleration of the disease and even moderate vision improvement can be achieved by anti-VEGF therapies [7]. The antagonist, either ranibizumab, aflibercept or the off-label used bevacizumab, is intravitreally injected. For best therapeutic outcome, injections need to be repeated on a monthly base [8]. Monthly intravitreal injections are a considerable burden for the patient and the executive clinics [9].

An important source for VEGF in the retina is the retinal pigment epithelium (RPE) [10], [11]. The RPE is an epithelial monolayer situated between the choroid and the photoreceptors. It has many functions which are necessary for upholding vision, such as nutrient supply, phagocytosis of shed photoreceptor fragments, recycling of visual pigment or the secretion of growth factors [12]. The RPE constitutively secretes VEGF towards the choroid as a protective factor and to uphold the fenestration of the choriocapillaries [11], [13], [14]. The secretion of VEGF can be elevated by many factors, such as oxidative stress or hypoxia [15]. The upregulation of VEGF by the RPE due to age-dependent or pathological alterations is considered an important factor in the development of wet AMD [16], [17].

Fucoidan is a complex sulfated polysaccharide extracted from brown algae which has been implicated to have anti-tumor, anti-oxidant and anti-inflammatory effects [18]–[22]. It is easily available from several marine algae species and is considered as functional food, which may exert systemic effects after oral administration. It has an excellent oral safety profile in animals and humans. Recently, it has been investigated in a clinical phase I and II study for the treatment of osteoarthritis [23]–[26]. Its anti-tumor properties have been suggested to be mediated by anti-angiogenic effects, which may be facilitated by interference of fucoidan with VEGF signaling [27], [28]. As these properties of fucoidan could also be beneficial in age-related macular degeneration, we were interested in the effects of fucoidan on RPE cells. In this study, we investigated the effects of fucoidan on RPE cells physiology, RPE- derived VEGF and RPE-induced angiogenesis.

## Materials and Methods

### Primary RPE isolation and culture

Porcine eyes were obtained with permission from the local abattoir (Fleischerei Loepthin, Jevenstedt, Germany), where the animals are killed for the purpose of food production and the eyes are regularly removed from the slaughtered animals due to legal regulations (Tier-LMHV (Anlage 5 zu §7 Satz 2, Kapitel III, Nr. 2.4). The usage of the eyes for experimental purposes was conducted in agreement with the animal welfare officer of the University of Kiel. According to the German animal welfare act (TierSchG), it is not considered to be animal research, but an alternative to the use of animals in research.

Primary porcine RPE cells are an established model and were isolated as previously described [29], [30]. The eyes were cleaned of adjacent tissue and immersed briefly in antiseptic solution. The anterior part of the eye was removed, as well as lens, vitreous and retina. In each eye cup, trypsin was added, and incubated for 5 min at 37°C. Trypsin solution was removed and substituted with trypsin-EDTA for 45 min at 37°C. RPE cells were gently pipetted off the choroid, collected in medium and washed. Cells were cultivated in Dulbecco's modified Eagle's medium (DMEM, PAA, Cölbe, Germany) supplemented with penicillin/streptomycin (1%), HEPES (25 mM), sodium-pyruvate (110 mg/ml) and 10% fetal calf serum (Linaris GmbH, Wertheim-Bettingen, Germany).

### ARPE-19 cell culture

ARPE-19 cells, an immortal human RPE cell line, were purchased from ATCC (Wesel, Germany) and cultivated in Dulbecco's modified Eagle's medium (DMEM; PAA,), supplemented with penicillin/streptomycin (1%), non-essential amino acids (1%), and 10% fetal calf serum (Linaris GmbH).

### Perfusion organ culture

Organ culture was prepared as previously described [31]. In brief, freshly slaughtered pig eyes were cleaned of adjacent tissue and immersed briefly in antiseptic solution. The anterior part of the eye was removed, RPE/choroid sheet were separated from sclera and the prepared tissue was fixed between the lower and upper part of a fixation ring. Organ sheets were cultivated in a perfusion chamber (Minucells & Minutissue, Bad Abbach, Germany). The chamber was placed on a heating plate and perfused with medium, (DMEM and Ham F12 medium (PAA) (1∶1) supplemented with penicillin/streptomycin (1%), HEPES (25 mM), sodium-pyruvate (110 mg/ml) and 10% porcine serum (PAA). The flow rate was 2 ml/hour. The gas exchange in this system takes place via silicone tubes; the pH and CO_2_ content of the media were stabilized by HEPES. The perfusion of the tissue allows a steady-state equilibrium of the tissue [32]. On the second day of cultivation, RPE/choroid sheets were exposed to fucoidan from *Fucus vesiculosus* (Sigma-Aldrich, Steinheim, Germany, Cat-Nr: F5631) (100 µg/ml) and the experiment was conducted as described elsewhere with modification [33]. In brief, supernatant was collected for one hour before treatment. Perfusion of the tissue was interrupted and the medium was transferred to a falcon tube where fucoidan was added. Additionally, fucoidan was added to the medium reservoir. The medium was transferred back into the chamber and the perfusion was restarted. For untreated cultures, the same procedure was conducted without addition of any substance. The supernatant was collected at designated time points (6 hours, 24 hours and 3 days) for one hour, centrifuged for 5 minutes at 13.000 rpm and stored at −20°C until further evaluation.

### MTT - assay

Cell viability in cell culture was tested on confluent cells with methyl thiazolyl tetrazolium (MTT) assay as described elsewhere [34] with modifications. In brief, MTT was solved 0.5 mg/ml in DMEM without phenol red (PAA). The cells were washed three times with PBS and incubated with MTT at 37°C for 2 hours. MTT was discarded and dimethyl sulfoxide (DMSO) was added to the cells. The tissue plates were shaken for 5 minutes, the DMSO collected and the absorption was measured at 550 nm with Elx800 (BioTek, Bad Friedrichshall, Germany).

### Trypan-blue exclusion assay (proliferation assay)

To determine the influence of fucoidan on proliferation, a defined number of ARPE-19 (500,000 cells) or primary porcine RPE cells (600,000 cells) were seeded on a 60 mm cell culture dish (Nunc, Roskilde, Denmark). One day after seeding, the cells were stimulated with 100 µg/ml fucoidan for 3 or 7 days. Cells were detached using trypsin/EDTA, centrifuged and resuspended in PBS. To determine the cell number, a trypan-blue exclusion assay was conducted as previously described [35].

### Scratch-assay

ARPE-19 cells, or porcine RPE-cells, were seeded in a 12-well-plate. Three wounds were scratched in the confluent cell layer with a toothpick and the cells were washed with PBS to remove detached cells. DMEM without phenolred supplemented with penicillin/streptomycin (1%), HEPES (25 mM), sodium-pyruvate (110 mg/ml), and 10% fetal calf serum was added, microscopic bright field pictures of three precise spots were taken and the coordinates were noted (Zeiss, Jena, Germany). Fucoidan (100 µg/ml) was added to the wells. 24 hours after application, another picture was taken at the same coordinates. To analyze the wound healing capability of the cells, application was conducted in duplicates and three pictures per well were taken. The gap size of the wound was measured with AxioVision Rel.4.8. (Zeiss, Jena, Germany), and the percentage of coverage of the wound was evaluated. Complete coverage was defined as 100%.

### Phagocytosis assay

Phagocytosis was assessed as previously described [36]. In brief, photoreceptor outer segments were prepared from porcine retina and used to opsonize FITC-labeled latex beads (diameter 1 µm). Opsonized beads were added to confluent primary RPE cells of 2^nd^ passage, treated with 100 µg/ml fucoidan for 1 hour, and incubated for 4 hour at 37°C. Cells were fixed and prepared for fluorescence microscopy. Eight pictures per slide were taken, beads and nuclei were counted, and the ratio determined.

### Treatment of cells for VEGF secretion

Confluent ARPE-19 cells were cultured with addition of 100 µg/ml fucoidan for 1, 3 and five days. Medium was changed and fucoidan added again at day 3 and 4 hours before collection Supernatant was collected, centrifuged for 5 minutes at 13.000 rpm and stored at −20°C until further evaluation.

### VEGF-ELISA

The VEGF-content of the supernatant of cell and organ cultures was measured by a VEGF-ELISA (R&D Systems, Wiesbaden, Germany) following the manufacturer's instructions. The range of detection of the ELISA was between 15 pg/ml and 1046 pg/ml. The ELISA detects all isoforms of VEGF-A, and readily detects porcine VEGF-A [29] as well as human VEGF-A.

### Immunocytochemistry

ARPE-19 or porcine RPE-cells, were seeded on coverslips (TH. Geyer, Hamburg, Germany), coated with Collagen A (Biochrom, Berlin, Germany). Confluent cells were exposed to fucoidan (100 µg/ml) for different time intervals. After incubation, cells were washed with PBS and fixed first in 6% PFA (Merck, Darmstadt, Germany), diluted in 2 x PEM-buffer (200 mM PIPES (Carl Roth GmbH, Karlsruhe, Germany), 2 mM magnesium chloride (Merck), 2 mM EGTA (Merck), pH 6.5) for 5 minutes. They were fixed in 6% PFA, diluted in 2 x borate-buffer (200 mM di-sodium tetraborate (Merck), 1.97 mM magnesium chloride, pH 11) for 10 minutes. The cells were permeabilized with 1% Triton X (Carl Roth GmbH) for 15 minutes and borohydride-solution was added to each well. After twofold washing with PBS, binding sites were blocked with Roti Immunoblock (Carl Roth GmbH) for at least one hour. Anti-VEGF (A-20) (Santa Cruz Biotechnology, Heidelberg, Germany, sc-152) as first antibody was dissolved in Roti Immunoblock, added and incubated over night at 4°C. Cells were washed with PBS three times, and the second antibody (Alexa Fluor 488 donkey anti-rabbit IgG (Invitrogen, Darmstadt, Germany)) with 0.4 µM bisbenzimide H in Roti Immunoblock was added. After washing with PBS and aqua dest., cover slides were mounted. As mounting medium, Slowfade gold antifade reagent (Invitrogen) was used. For analyzing, stained cells were visualized with Axio Imager Z1 (Zeiss, Jena, Germany).

### Treatment of cells for Western blotting

In order to determine the influence of fucoidan on VEGF in the presence of bevacizumab, confluent ARPE-19 cells were stimulated with 250 µg/ml bevacizumab and 100 µg/ml fucoidan for 1 day, 5 days and 7 days.

### Whole cell lysate

Whole cell lysates of ARPE-19 cells after treatment were prepared in an NP-40 buffer. For this, cells were scraped off in PBS, centrifuged, and the pellet was resuspended in in NP-40 buffer (1% Nonidet P40 Substitute (Fluka, Steinheim, Germany), 150 mM NaCl (Carl Roth GmbH), 50 mM Tris (Sigma-Aldrich), pH 8,0) and lysed on ice for at least 30 minutes. Samples were centrifuged at 13.000 rpm for 15 minutes. The protein concentration of the supernatant was determined by BioRad protein assay with BSA as standard.

### Western blot

Western blot to detect VEGF expression was conducted as described elsewhere with modifications [30]. To separate proteins with SDS-PAGE, a resolving gel with 12% acrylamide was used. After blotting the gel, the PVDF-membrane (Carl Roth GmbH) was blocked with 4% skim milk in Tris buffered saline with 0.1% Tween for 1 hour at room temperature. The blot was treated with the first antibodies, against beta-actin (Cell Signaling Technologies) or VEGF (A-20) (Santa Cruz Biotechnology), overnight at 4°C in 2% skim milk in Tris buffered saline with 0.1% Tween. The VEGF antibody used detects intracellular VEGF containing a signal peptide which initiates export across the endoplasmic reticulum; this signal peptide is cleaved before secretion. After washing, the blot was incubated with anti-rabbit IgG, HRP-linked Antibody (Cell Signaling Technologies) in 2% skim milk in Tris buffered saline with 0.1% Tween. Following the final washing, the blot was incubated with Immobilon chemiluminescence reagent (Millipore, Schwalbach, Germany) and the signal was detected with MF-ChemiBis 1.6 (Biostep, Jahnsdorf, Germany). The density of the bands was evaluated using Total lab software (Biostep) and the signal was normalized for β-actin.

### Isolation of outgrowth endothelial cells from the peripheral blood

Outgrowth endothelial cells are endothelial cells which can be isolated from peripheral blood in high purity in terms of endothelial cell markers. These cells were isolated from buffy coats and characterized as previously described [37], [38]. In brief, blood mononuclear cells were isolated by Biocoll (Biochrom, Berlin, Germany) density centrifugation. Mononuclear cells were seeded onto collagen coated 24-well plates in a density of 5×10^6^ cells/well in EGM-2 (Lonza, Belgium) with full supplements from the kit, 5% FBS (PAA Laboratories, Pasching, Austria), and 1% penicillin/streptomycin (PAA Laboratories). After one week, adherent cells were collected by trypsin and reseeded on collagen coated 24-well plates in a density of 0.6×10^6^ cells/well. After 2–3 weeks, colonies of endothelial cells (OEC) were harvested and further expanded over several passages using EGM-2 in a splitting ratio of 1∶2.

### Matrigel angiogenese assay and viability assessment

Angiogenesis experiments were performed on Ibidi `Angiogenesis slides by placing 10 µl of matrigel diluted 1∶1 in EGM-2 without VEGF in the inner well of the IBIDI slide. After gelation at 37°C for 30 minutes, 10.000 cell OEC/well were seeded in volume of 50 µl EGM-2 (without VEGF) containing the following factors: a) 50 ng/ml VEGF b) 50 ng/ml VEGF plus 100 µg/ml fucoidan c) 100 µg/ml fucoidan d) conditioned medium from retinal pigment epithelial cells donor 1 (RPE1) e) conditioned medium from retinal pigment epithelial cells donor 2 (RPE2) f) conditioned medium from RPE1 plus fucoidan (100 µg/ml), e) conditioned medium from RPE2 plus fucoidan (100 µg/ml) and g) EGM-2 containing all supplements from the kit besides VEGF.

After 1 day of culture, cells were analysed for angiogenic activity after the treatment with respective substances as described above. In addition, the cellular viability was assessed using calcein-AM. For this purpose, cells were treated with 0.2 µg/ml calcein-AM (BD, Heidelberg, Germany) in cell culture medium for 10 minutes. After medium exchange, cells grown on the matrigel substrate were visualized on a confocal laser scanning microscope (Zeiss LSM 510 Meta, Jena, Germany). For each treatment, at least 3 pictures were taken from two technical replicates. These experiments and the picture analysis were performed with endothelial cells from three different donors.

### Image Analysis

The microscopic images were analyzed using the image processing program ImageJ Vers. 1.47 [39]. In brief, tube-like structures were extracted from the background by automatic segmentation after background correction. The binaries of the tube-like structures were further processed, including smoothing and a final manual correction. The resulting binaries were processed in several steps, yielding a skeleton as previously described [40] and the quantitative analysis of skeleton-length was used to characterize the tubular structures.

### Statistics

Statistical analysis was performed with MS-Excel. Means ± standard deviation (s.d.) was calculated for at least 3 independent sets of experiments. Significant differences between means were calculated by an unpaired t-test. A p-value of 0.05 or less was considered significant. For angiogenesis assay, images from 3 donors were quantified and means ± s.d. were calculated for each treatment (n = 9 to 14). Significant differences between means were calculated by an unpaired t-test for either homoscedastic or heteroscedastic variances according to the results of a previous variance ratio analysis (F-test, p<0.05).

## Results

### Toxicity of fucoidan

**Figure 1:**
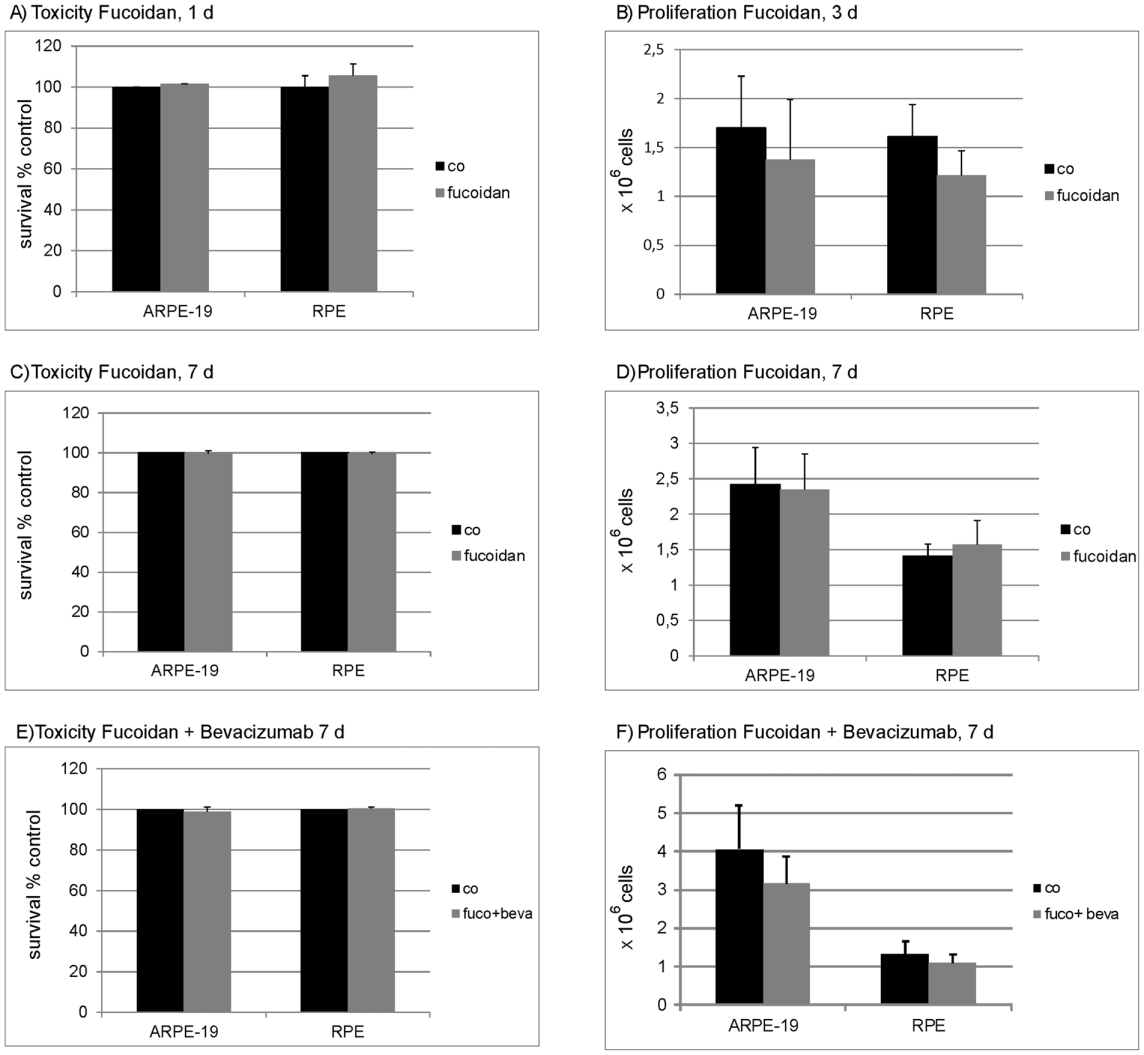
Toxicity and proliferation. To investigate toxicity, RPE or ARPE-19 cells were treated with 100 µg/ml fucoidan for 24 hours (A) or 7 days (C). In addition, cells were treated with a combination of fucoidan (100 µg/ml) and bevacizumab (250 µg/ml) for 7 days (E). Toxicity was measured with MTT test. Fucoidan did not exert toxic effects on RPE or ARPE-19 cells at any tested application (A,C,E). To investigate proliferation, a defined number of cells were seeded, cells were treated with fucoidan (100 µg/ml) and cell number was assessed after 3 days (B) and 7 days (D). In addition, cells were treated with a combination of fucoidan (100 µg/ml) and bevacizumab (250 µg/ml) and cell number was assessed after 7 days (F). No significant influence on proliferation was found. Significance was determined with student's t-test. Co  =  untreated control, fuco  =  fucoidan, beva  =  bevacizumab.

Toxicity of fucoidan was tested in MTT assay. No toxicity of fucoidan (100 µg/ml) applied for 24 hours could be detected in ARPE-19 (101.5% (±5.52)) or in porcine primary RPE cells (105.6% (±5.50)) (Fig. 1A). Similar results were obtained after 7 days, with no toxicity detected in ARPE-19 cells (99.72% (±1.36)) and in primary porcine RPE cells (99.38% (± 0.93)) (Fig. 1C). Additionally, the toxicity of a combined treatment with fucoidan and bevacizumab after seven days was assessed. No toxicity could be observed (ARPE-19: 98.7% (±2.25); RPE: 100.47% (±0.55)) (Fig. 1E).

### Influence of fucoidan on proliferation

To analyze the effect of fucoidan on proliferation, definite cell numbers of ARPE-19, or porcine RPE cells, were seeded and the cell number was assessed with a trypan blue exclusion assay after 3 days or 7 days of incubation. No statistical significant effect on proliferation was found for either cell type after 3 days (ARPE-19: control 1.46×10^6^ cells (±0.77); fucoidan 1.44×10^6^ cells (±0.25); RPE: control 1.61×10^6^ cells (±0.32); fucoidan 1.21×10^6^ cells (±0.28)) (Fig. 1B), or after 7 days (ARPE-19: control 2.42×10^6^ cells (±0.52); fucoidan 2.35×10^6^ cells (±0.50); RPE: control 1.41×10^6^ cells (±0.17); fucoidan 1.57×10^6^ cells (±0.34)) (Fig. 1D). In addition, the effect of a combined treatment with fucoidan and bevacizumab on proliferation was assessed. After 7 days of combined treatment, no statistical significant effect on proliferation was found for either cell type (ARPE-19: control 4.05×10^6^ cells (±1.17), fucoidan and bevacizumab: 3.16×10^6^ cells (±0.72); RPE: control 1.32×10^6^ cells (±0.32), fucoidan and bevacizumab 1.09×10^6^ cells (±0.21)) (Fig. 1F).

### Influence of fucoidan on phagocytosis

**Figure 2:**
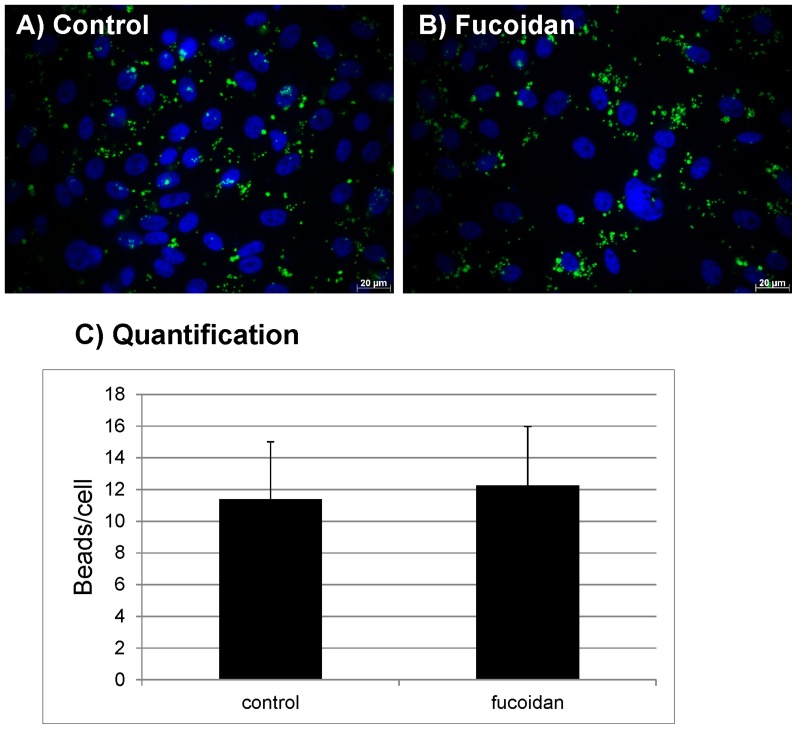
Phagocytosis. Primary RPE cells were stimulated with 100 µg/ml fucoidan for 1 hour. RPE cells were exposed to FITC-labeled, photoreceptor outer segment opsonized beads for 4 hours and uptake of the beads was evaluated in fluorescence microscope. No influence of fucoidan on RPE phagocytosis was found. A) control, B) fucoidan, C) quantification of uptaken beads. Significance was determined with student's t-test.

To analyze the effect of fucoidan on the phagocytosis of photoreceptor outer segments by primary RPE, a phagocytosis assay using POS-opsonized beads was conducted, which detects bound and internalized beads. No influence of fucoidan on the phagocytosis by the RPE could be found (control: 11.38 ± 3.63 beads/cells; 100 µg/ml fucoidan: 12.24±3.72 beads/cell) (Fig. 2).

### Influence of fucoidan on wound healing

**Figure 3:**
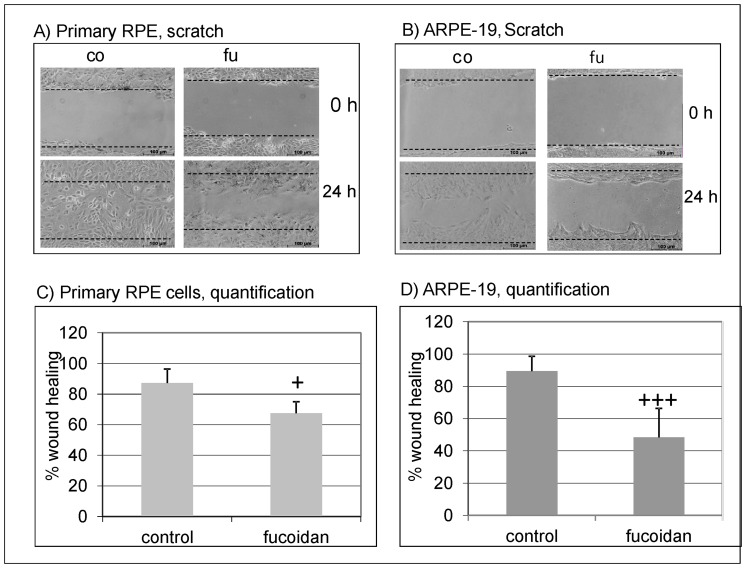
Wound healing. A wound was scratched in a confluent cell layer of primary porcine RPE cells and ARPE-19 cells. Cells were either untreated (control) or exposed to fucoidan (100 µg/ml) for 24 hours. Exemplary pictures of wound healing are depicted for primary RPE cells (A) and ARPE-19 cells (B). The percentage of coverage after 24 hours of wound healing is depicted in the graphs for primary RPE cells (C) and ARPE-19 cells (D). Fucoidan significantly reduces wound healing in both RPE and ARPE-19 cells. Significance was determined with student's t-test, + p<0.05; +++ p<0.001.

To analyze the effect of fucoidan on wound healing, a scratch assay was performed with ARPE-19 and primary porcine RPE cells. In untreated primary RPE cells, 87.30% (±9.05) of the wound was closed after 24 hours. In contrast, in primary RPE cells treated with fucoidan, only 67.47% (±7.56) of the wound was closed. Similar results were obtained in ARPE-19 cells, where in the untreated control, 87.23% (±8.7) of the wound was covered in contrast to fucoidan stimulated cells, where only 41.24% (±9.54) of the area was covered (Fig. 3).

### Influence of fucoidan on VEGF secretion

**Figure 4:**
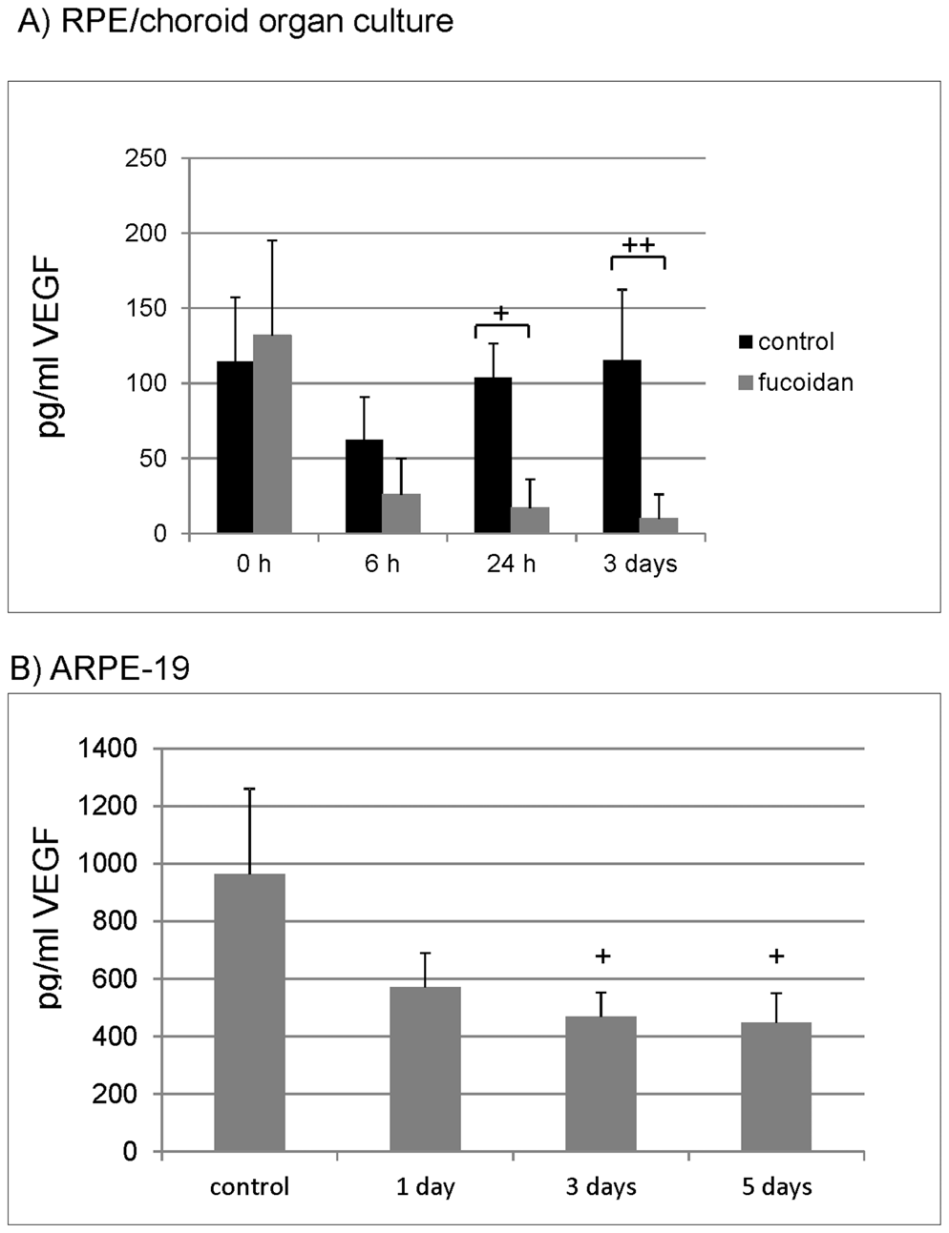
VEGF secretion. VEGF secretion was investigated in RPE/choroid organ culture (A) and ARPE-19 cell culture (B). RPE/choroid perfusion organ cultures were treated with 100 µg/ml fucoidan for 3 days and supernatant was collected at 6 hours, 24 hours and 3 days for one hour. ARPE-19 cells were treated with 100 µg/ml fucoidan for five days, and medium was collected after 1 day, 3 days and 5 days. VEGF content was evaluated with ELISA. Fucoidan reduced VEGF content compared to control in organ culture after 24 hours and 3 days (A). In cell culture, a reduction of VEGF secretion can be found after 3 days and 5 days (B). Significance was determined with student's t-test, + p<0.05; ++ p<0.01.

The effect of fucoidan on the secretion of VEGF was tested in ARPE-19 cells and RPE/choroid perfusion organ culture. Supernatant of cell and organ cultures was evaluated for VEGF content in VEGF ELISA. In RPE/choroid organ culture, VEGF reduction was found after 6 hours, and reduction reached significance 1 day and 3 days post stimulus compared to untreated control (6 hours: control 62.40±28.29 pg/ml, fucoidan 25.90%±24.00, p = 0.16; 1 day: control 103.76±22.80 pg/ml, fucoidan 16.91±19.09), p<0.01; 3 days: control 115.35±47.00 pg/ml, fucoidan 9.53±16.51, p<0.05) (Fig. 4A). In ARPE-19 cell culture, the secretion of VEGF was reduced compared to control after 1 day (earliest time point tested), and the reduction was significant at day 3 and day 5 (control: 965.45 ± 295.21 pg/ml VEGF; 1 day: 571.26. ±118.52, p = 0.098; 3 days: 469.48, ±82.83., p<0.05; 5 days: 447.92±102.02, p<0.05)) (Fig. 4B).

### Influence of fucoidan on VEGF expression

**Figure 5:**
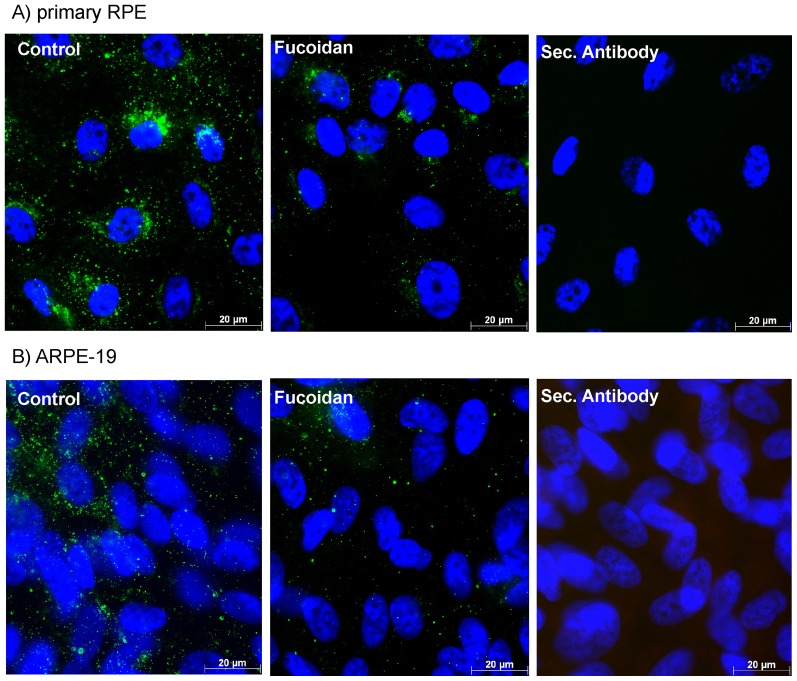
VEGF165 expression. Primary RPE cells (A) and ARPE-19 cells (B) were treated with 100 µg/ml fucoidan for 24 hours and the expression of intracellular VEGF (still containing signal peptide) was evaluated using immunocytochemistry. Cells treated with fucoidan exhibited a substantial decrease in intracellular VEGF expression.

Confluent ARPE-19 or primary RPE cells were exposed to fucoidan (100 µg/ml) for 3 days, and the expression of intracellular VEGF was detected in immunohistochemistry. A clear reduction of the intracellular VEGF signal is seen in cells stimulated with fucoidan (Fig. 5).

### Influence of fucoidan on VEGF expression in the presence of bevacizumab

**Figure 6:**
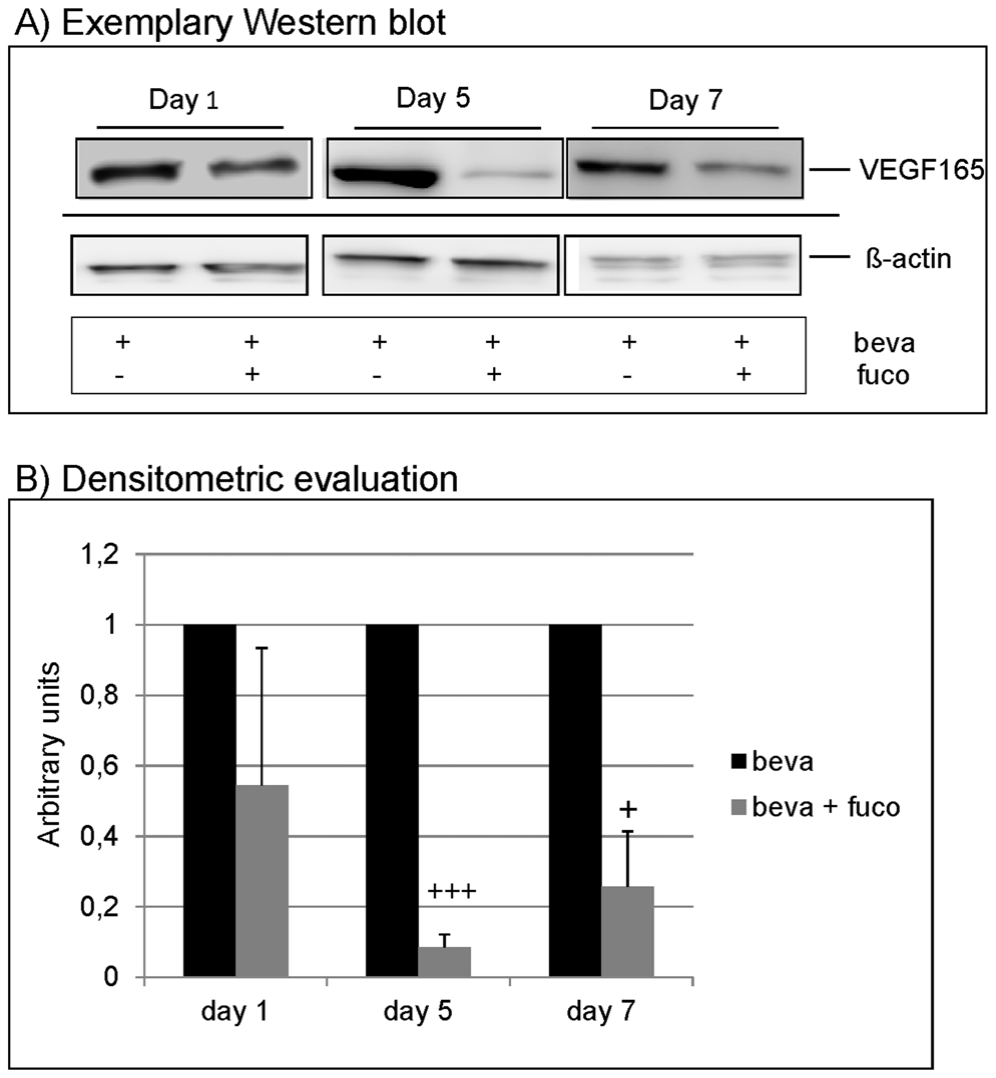
VEGF expression in presence of bevacizumab. ARPE-19 cells were treated with 250 µg/ml bevacizumab and 100 µg/ml fucoidan for 1 day, 5 days and 7 days. Controls cells were treated with bevacizumab only. Western blot for intracellular VEGF (still containing signal peptide) depicted a reduction of VEGF165 in Western blot at day 5 and day 7 (A), which was significant in densitrometric evaluation (B). Significance was determined with student's t-test, + p<0.05; +++ p<0.001. beva  =  bevacizumab, fuco  =  fucoidan.

Bevacizumab is an anti-VEGF antibody commonly used in anti-VEGF therapy. In order to evaluate whether fucoidan also exerts effects on VEGF expression in cells treated with a VEGF antagonist, ARPE-19 cells were stimulated with the clinically relevant concentration of bevacizumab (250 µg/ml) and fucoidan (100 µg/ml) for 1 day, 5 days and 7 days, evaluating the effect on VEGF expression in Western blot. After one day of application, a slight reduction of VEGF165 expression can be found (Fig. 6A). After 5 and 7 days of incubation, a strong reduction was seen (Fig. 6A) that reached significance in densitometric evaluation (day 1: 0.544±0.39; day 5: 0.085±0.036, p<0.001; day 7: 0.256±0.16, p<0.05) (Fig. 6B).

### Influence of fucoidan on angiogenesis

**Figure 7:**
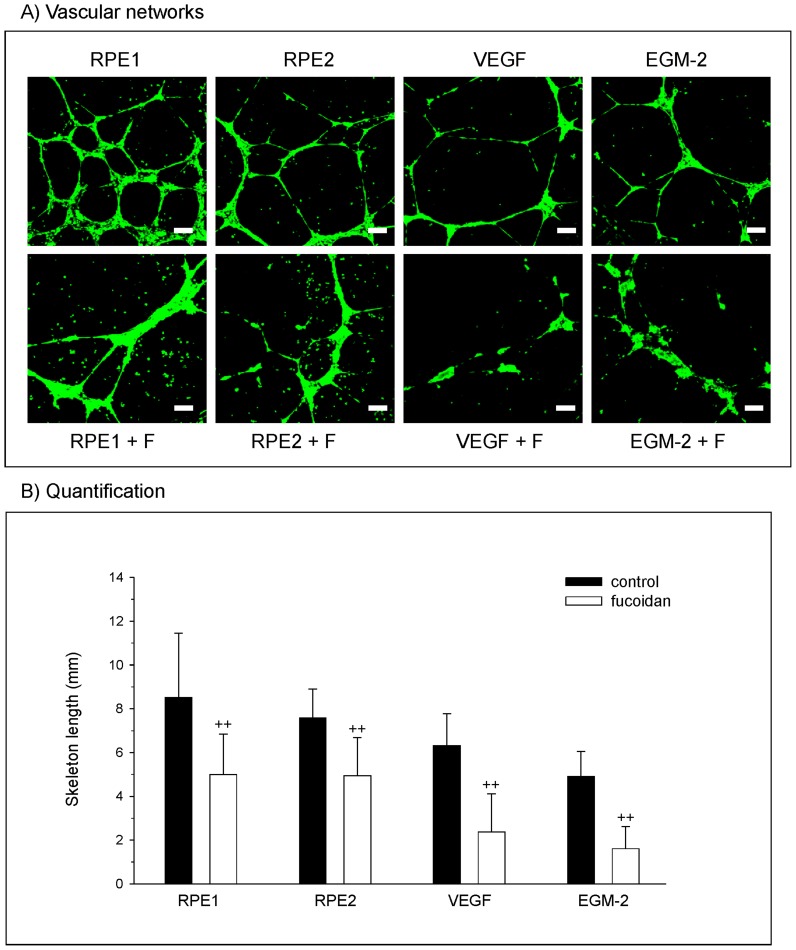
Angiogenesis. (A) Morphological appearance of OEC grown on Matrigel and stained with Calcein-AM which is converted to a green fluorescence by viable cells. Results indicated angiogenic structures in OEC treated with conditioned medium from RPE cells from different donors, VEGF and the EGM-2 (no VEGF). Additional treatment with fucoidan resulted in the reduction of vascular structures. (B) Quantitative image analysis depicting the skeleton length of angiogenic structures. Significance was determined with student's t-test, ++ p<0.01.

On the matrigel matrix outgrowth, endothelial cells formed interconnected vascular networks. These networks were increased in the presence of conditioned medium from the retinal pigment epithelium cells (Fig. 7A), RPE1 and RPE 2, in comparison to the positive control for angiogenesis including a concentration of 50 ng/ml VEGF. In EGM-2, which contained all growth factors provided by the bullet kit with the exception of VEGF, OEC showed some angiogenic structures. Nevertheless, in all approaches, the addition of fucoidan resulted in a reduction of angiogenic structures, whereas the cells were still viable as indicated by the staining for the viability marker calcein-AM. According to these morphological observations, images derived from experiments of three different donors of OEC were analysed by quantitative image analysis to quantify the anti-angiogenic effect of fucoidan. The results are depicted in Fig. 7B using the skeleton length of vascular structures as indicator for the angiogenic activity. Quantitative evaluation indicated a statistical significant reduction of the skeleton length in the presence of fucoidan in all groups tested.

## Discussion

In our study, we investigated the effects of fucoidan on RPE cells, utilizing three different models of RPE cells: ARPE-19 cells, primary porcine cells of second and third passage, and RPE/choroid organ culture. While the use of ARPE-19 cells as a model of RPE cells is under debate [41], it is still considered a valuable tool in RPE research. However, data obtained with ARPE-19 cells should be confirmed using models which resemble natural RPE cells more closely. In our study, we have used primary RPE cells of second and third passage, which display a cobble-stone morphology and are still pigmented, indicating a high differentiation. Moreover, in RPE/choroid organ cultures, RPE cells are cultured on their natural substrate, the Bruch's membrane, with connections to the choroid. A constant perfusion generates a steady state equilibrium. RPE cells in this culture maintain their morphology and differentiation for the time period investigated [31].

The rationale of this study was to conduct first line in vitro experiments to test whether fucoidan might be a possible candidate for further investigation for the treatment of AMD. Fucoidan is a complex, heterogeneous mixture of branched, sulfated polysaccharides found in brown algae and marine organisms [18]. Many studies have shown a variety of beneficial effects of this polysaccharide, such as anti-inflammatory, anti-tumor, anti-oxidative and even complement inhibiting properties [18]–[22]. As the current concepts of AMD development include oxidative stress, complement activation and inflammatory events, fucoidan may be an interesting molecule to be studied for possible AMD intervention. Furthermore, fucoidan has been described to be anti-angiogenic, possibly by inhibiting VEGFR-2 signal transduction [27], [28]. As VEGF is the current treatment target for the therapy of AMD, we focused on its effects on VEGF derived from the retinal pigment epithelium.

First, we assessed the effect of fucoidan on the physiology of RPE cells. We tested toxicity, proliferation, wound healing and phagocytosis. Fucoidan has been described to exert apoptotic effects on neoplastic cells [42], [43], but no general toxicity has been found so far [23]–[26]. In accordance with this, no toxicity of fucoidan on RPE cells, both primary and cell line, could be detected in our study. In addition, no toxicity on endothelial cells could be detected at the investigated time points. Furthermore, RPE cell proliferation was not altered, in contrast to the effect of fucoidan on neoplastic cells [42], [44]. An important task of RPE cells is the phagocytosis of photoreceptor outer segment. Fucoidan has previously been shown to interfere with phagocytotic functions [45]. However, we did not find any reduction of phagocytosis of photoreceptor outer segments in fucoidan treated cells, suggesting that no interference with this function occurred. The only effect of fucoidan on RPE cell physiology found in our study was a decline in wound healing ability assessed by scratch assay. As we did not find any influence of RPE cell proliferation, this reduction is most likely due to an impairment of migration. Fucoidan is the binding partner of several extracellular matrix interacting molecules such as integrins [46], which may provide an explanation for a reduced migratory ability. As RPE cells are generally post mitotic and do not migrate in a physiological situation, this property of fucoidan should not be of further consequence in the retina. However, therapeutic laser burns in the retina may be covered by migrating RPE cells [47], so fucoidan may interfere with wound healing after laser treatment. Furthermore, coverage of small RPE lesions or small RPE tears may be disturbed when migration is inhibited by fucoidan [48], [49]. In dry AMD, however, RPE migration in the retina has been observed in a high percentage of patients, which is found especially over drusen [50]. This might also be suppressed by fucoidan, thus possibly reducing AMD-related anatomical changes. Taken together, a general excellent toxicity profile can be confirmed for RPE cells.

The main topic of this study was the effect of fucoidan on RPE derived VEGF. We were able to show that fucoidan reduces VEGF secretion in RPE cells and RPE/choroid organ cultures. In addition, fucoidan reduces intracellular VEGF expression in RPE cells. These data indicate a possible use as a VEGF-antagonist. Moreover, even when bevacizumab is present, fucoidan further reduces VEGF expression, indicating that fucoidan may exert additional beneficial effects even under anti-VEGF treatment and may be useful as an additive therapy. Finally, we were able to show that fucoidan reduces angiogenesis induced by RPE supernatant as well as by VEGF alone, which is in concordance with the published anti-angiogenic effects of fucoidan [27], [51]. This shows that the anti-angiogenic effect is not only found in a neoplastic context but is also valid for RPE-induced angiogenesis.

The pathways through which fucoidan reduces VEGF expression and secretion are not known. Fucoidan is able to bind to VEGF165 and reduce VEGFR-2 signaling [27], [28]. In previous studies, we could show that extracellular inhibition of VEGF reduces VEGF expression in RPE cells [30] and that inhibition of VEGFR-2 reduces VEGF secretion in RPE organ culture [33], indicating positive autocrine regulatory effects of VEGF. Thus, a possible pathway through which fucoidan reduces VEGF expression may be the inhibition of autocrine VEGFR-2 signaling. However, fucoidan reduces VEGF expression even at a concomitant application with bevacizumab. As bevacizumab at the concentrations used is able to bind to all available extracellular VEGF [30], the inhibition of an autocrine positive feedback loop cannot be the only mechanism of VEGF reduction. The exact pathways of fucoidan mediated VEGF reduction needs to be further elucidated.

Fucoidan is currently considered a functional food, but is also investigated in clinical trials [23], [52]. Its effects have been studied not only in vitro, but also in animal and human studies, were it exhibits an excellent toxic profile [23]–[26]. While its oral availability is under debate [22], recent studies indicate a possible absorption of fucoidan by the gastrointestinal tract [18], [26], which would render an oral application an attractive alternative to intravitreal injections. However, our data, obtained in vitro, need to be confirmed in vivo in order to elucidate its possible transferability into the living organism.

In conclusion, we show that fucoidan is safe for RPE cells and reduces VEGF expression and secretion in RPE cells, as well as VEGF-induced angiogenesis, making it an interesting molecule for further studies for the use in AMD.
